# A framework for automated enrichment of functionally significant inverted repeats in whole genomes

**DOI:** 10.1186/1471-2105-11-S6-S20

**Published:** 2010-10-07

**Authors:** Cyriac Kandoth, Fikret Ercal, Ronald L Frank

**Affiliations:** 1 Department of Computer Science, Missouri University of Science and Technology, Rolla MO, 65409, USA; 2 Department of Biological Sciences, Missouri University of Science and Technology, Rolla MO, 65409, USA

## Abstract

**Background:**

RNA transcripts from genomic sequences showing dyad symmetry typically adopt hairpin-like, cloverleaf, or similar structures that act as recognition sites for proteins. Such structures often are the precursors of non-coding RNA (ncRNA) sequences like microRNA (miRNA) and small-interfering RNA (siRNA) that have recently garnered more functional significance than in the past. Genomic DNA contains hundreds of thousands of such inverted repeats (IRs) with varying degrees of symmetry. But by collecting statistically significant information from a known set of ncRNA, we can sort these IRs into those that are likely to be functional.

**Results:**

A novel method was developed to scan genomic DNA for partially symmetric inverted repeats and the resulting set was further refined to match miRNA precursors (pre-miRNA) with respect to their density of symmetry, statistical probability of the symmetry, length of stems in the predicted hairpin secondary structure, and the GC content of the stems. This method was applied on the *Arabidopsis thaliana* genome and validated against the set of 190 known Arabidopsis pre-miRNA in the miRBase database. A preliminary scan for IRs identified 186 of the known pre-miRNA but with 714700 pre-miRNA candidates. This large number of IRs was further refined to 483908 candidates with 183 pre-miRNA identified and further still to 165371 candidates with 171 pre-miRNA identified (i.e. with 90% of the known pre-miRNA retained).

**Conclusions:**

165371 candidates for potentially functional miRNA is still too large a set to warrant wet lab analyses, such as northern blotting, on all of them. Hence additional filters are needed to further refine the number of candidates while still retaining most of the known miRNA. These include detection of promoters and terminators, homology analyses, location of candidate relative to coding regions, and better secondary structure prediction algorithms. The software developed is designed to easily accommodate such additional filters with a minimal experience in Perl.

## Background

In the last decade, non-coding RNA (ncRNA) sequences have become more essential to our understanding of gene organization. They were once considered insignificant in comparison to protein-coding sequences. But since then, a variety of new types of ncRNA genes have been discovered, each of them revealing new biological roles and cellular mechanisms like gene silencing, replication, gene expression regulation, transcription, chromosome stability, and protein stability [1-3]. Therefore, the identification of ncRNA has significant importance to the biological and medical community. To date, the genomes of numerous organisms have been fully sequenced, making it possible to perform genome-wide computational analyses. Computational methods of ncRNA identification typically involve scanning genomic DNA or transcriptome data for candidate sequences, after which wet lab techniques like northern blotting are required to verify their cellular function [4].

The precursors of non-coding RNA sequences like transfer RNA, ribosomal RNA, microRNA, and small-interfering RNA, typically adopt hairpin-like, cloverleaf, or similar symmetric structures which are the result of dyad symmetry, i.e. inverted repeats (IRs) in the RNA sequences. But hundreds of thousands of IRs that can be found by a simple scan of genomic DNA. This makes it difficult to claim that any inverted repeat in a genome has functional significance, but it potentially raises the number of functional RNA sequences that have yet to be identified.

**Figure 1:**
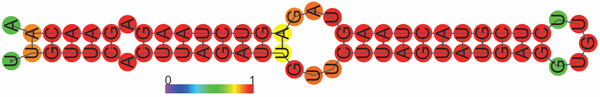
**A typical hairpin-like secondary structure of a microRNAprecursor.** This secondary structure was generated using the RNAfold secondary structure predictor of the Vienna RNA WebSuite on a known Arabidopsis microRNA precursor retrieved from the miRBase database [miRBase:MI0008304]. The color-code used represents the base-pair probabilities based on a minimum free energy analysis.

In this paper, we focus on the identification of microRNAs (miRNA) which are short, ~22 nucleotide long ncRNAs that are involved in gene regulation post-transcription. This can occur through cleavage of the messenger RNA, or through translational repression causing regulation of a specific protein [5]. The processing of miRNA from genomic DNA and its subsequent activation in cells is a multistep process that starts with transcription from genomic DNA into RNA transcripts called primary miRNAs (pri-miRNAs). These variable length transcripts contain the mature miRNA as a subsequence, with inverted repeats that usually form a more stable hairpin-like structure called a precursor-miRNA (pre-miRNA). This structure can range from 53 to 215 base-pairs long in animals, and more variable lengths among plants, with long stems, and relatively small loops [6]. A sample pre-miRNA hairpin structure is shown in Figure 1. The pre-miRNA hairpin is released from the pri-miRNA transcript with the help of ribonuclease Drosha [7]. Recently, a type of miRNA that bypasses Drosha processing has been discovered [8], but most known miRNAs are still subject to processing by Drosha. After the pre-miRNA hairpin is released, it is exported from the cell nucleus to the cytoplasm where the ribonuclease Dicer cleaves the pre-miRNA approximately 19 bp from the Drosha cut site resulting in a double-stranded RNA. One of these two strands becomes the mature miRNA sequence by associating itself with the RNA-Induced Silencing Complex (RISC). RISC uses the miRNA as a template for recognizing complementary target messenger RNA (mRNA) to regulate a specific protein coding gene. Several miRNA identification strategies take advantage of this understanding of miRNA processing and activation and they are discussed below.

Transcription from DNA to RNA is typically guided by the presence of promoter and terminator sequences in the genome that usually lie in the vicinity of non-coding or protein coding genes. However, current methods can only detect certain classes of promoters and terminators, and the degrees of accuracy of such methods are insufficient for genome-wide scans [9]. In addition to this, the starting points of the transcripts in the genome are not always known, even for commonly studied genes. It has been reported that some intergenic regions (DNA between protein coding genes) contain ncRNA that act to regulate the genes nearby. Hence, many RNA detection methods make the assumption that ncRNA is present in the vicinity of known genes and between coding regions within genes (introns). However, most intergenic DNA still have no known function and the basis for this assumption is anecdotal. Current estimates show that approximately 60% of miRNAs are expressed independently, 15% of miRNAs are expressed in clusters, and 25% are in introns [10].

If an RNA is functionally significant, then the structure and sequence are conserved over the generations. A method called miRNAminer [11] searches for such evolutionarily related miRNA sequences from different species (homologs). Given a query miRNA, candidate homologs from different species are tested for secondary structure, alignment and conservation, in order to assess their candidacy as miRNAs. By computationally identifying small sections of a genome that could form hairpin-like secondary structures, some researchers have been able to identify sets of potential miRNA sequences which include a subset of known miRNA. Two such methods, miRSeeker [12] and miRScan [10], first identify hairpin structures from intergenic regions using homology search and secondary structure prediction. To these candidates, miRSeeker applies mutation patterns that are typical of miRNAs, and miRScan identifies those structures having features such as symmetric bulges or a highly conserved stem near the terminal loop. miRRim [13] represents the evolutionary and secondary structural features of all known miRNA and their surrounding regions with a sequence of multidimensional vectors. It uses these to train hidden Markov models (HMM) for miRNA and non-miRNA sequences. These models are combined into a single HMM and used to search genomic sequences for miRNA. Current methods of secondary structure prediction involve a dynamic programming algorithm similar to those used for sequence alignment. These methods are promising, but cannot predict more complex secondary structures like pseudoknots (non-nested pairing). Clote et al [14] proposed that the secondary structures of functional ncRNA are more thermodynamically stable than random RNA. The Gibbs free energy (∆G°) is a popularly used measure of this thermodynamic potential energy, and some ncRNA detection methods incorporate it as a threshold for detection of miRNA [11].

The hairpin-like secondary structure of a microRNA is a result of the inverted repeats that it contains. It is believed that IRs are the result of inverted DNA duplication events that occurred during the course of evolution of most organisms [15]. If this is the case, the asymmetries and bulges as seen in Figure 1 are formed later as a result of accumulation of mutations, insertions, and deletions. However, the inverted repeats remain highly conserved since the base-paired stem loops of the hairpin structures are relatively much longer than the asymmetries. The degree of dyad symmetry can therefore be used as a criterion for miRNA detection. We present a fast genome-wide scanning algorithm named irScan that first finds all sufficiently symmetric IRs in a given genomic DNA sequence (typically a whole chromosome). This large number of ncRNA candidates is then further reduced based on user-defined criteria for ncRNA detection. We demonstrate the capability of this algorithm using criteria for miRNA detection like the density of symmetric matches in the IR (density of base-pairs in the hairpin-like secondary structure), statistical probability of the symmetry, average length of contiguous symmetric matches in the IR (length of base-paired stems in the hairpin), and the GC content of the matches in the IRs. Detection of inverted repeats by itself is an insufficient criterion for ncRNA detection. Our preliminary scan using irScan's base thresholds on the fully sequenced Arabidopsis chromosomes revealed around 1.1 million mostly symmetric IRs. It is thus necessary to bring this number down to a small set of candidates that are most likely to be functionally significant ncRNA and hence warrant further wet lab analyses.

## Methods

### Detection of inverted repeats

irScan starts by scanning for IRs in the given genomic sequence using a variation of the Smith-Waterman (SW) local alignment algorithm [16]. The original SW algorithm is a dynamic programming technique that generates an optimal gapped local alignment between two given sequences based on a predefined scoring matrix for matches, mismatches, and gaps. An implementation of this algorithm was written in C++ that takes only one sequence as input, translates the DNA character set (ACGT) to the RNA character set (ACGU), generates a reverse complement of it, and then aligns it against the original sequence. The resulting local alignment would then reveal an optimal inverted repeat in the original sequence based on the match and mismatch penalties shown in Table 1, and a gap penalty of -6. These penalties appeared to work best at predicting the secondary structures of the known pre-miRNA sequences. Since this algorithm returns only one IR per input sequence, longer input sequences need to be subdivided further to detect shorter clustered IRs. So irScan used scanning windows of sizes 600, 300, and 150 base pairs to reflect the various sizes of known pre-miRNA. Each scanning window skips through the given genomic sequence by half the number of base pairs i.e. a skip size of 300bp is used for the 600bp scanning windows, 150bp skip size for 300bp windows, and 75bp for 150bp windows. The shorter of the matching IRs (duplicates) generated by adjacent overlapping windows of the same size are removed. But the duplicates generated by overlapping windows of different sizes are retained because the duplicate removal process cannot distinguish between nearby identical IRs and duplicate IRs generated by overlapping scanning windows. However, the benefit of substantial reduction in the number of IRs using this simple duplicate remover outweighed the computational cost of implementing a more accurate but complex duplicate remover. For all runs of irScan, the simpler duplicate removal process was implemented to considerably reduce the number of pre-miRNA IR candidates, but only after all the miRNA specific filters were applied. These filters are explained below.

**Table 1:** Scoring matrix used by irScan's IR detector

	A	C	G	U	N
**A**	5	-4	-4	-4	0
**C**	-4	5	-4	-4	0
**G**	-2	-4	5	-4	0
**U**	-4	-2	-4	5	0
**N**	0	0	0	0	3

The local alignment scoring matrix used when aligning a nucleotide sequence against its reverse complement. Matches score 5 points, while mismatches are penalized by 4. All unresolved ambiguous loci are treated as an N regardless of which bases are more probable. This matrix appears to work best at predicting the secondary structures of the known 190 pre-miRNA. Note that G-A and U-C mismatches are not penalized as much to accommodate the occurrence of G-U and U-G base pairs respectively.

### MicroRNA precursor analysis

**Figure 2:**
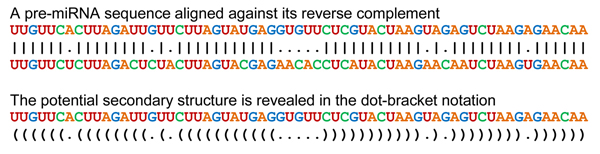
**irScan's IR detector emulates a secondary structure predictor.** The variant of the Smith-Waterman algorithm used by irScan's IR detector generates this dot-bracket notation of the secondary structure when used on a known Arabidopsis precursor [miRBase:MI0008304]. The inverted repeats form 25 base pairs of which two are weaker G-U matches surrounded by matches.

**Figure 3:**
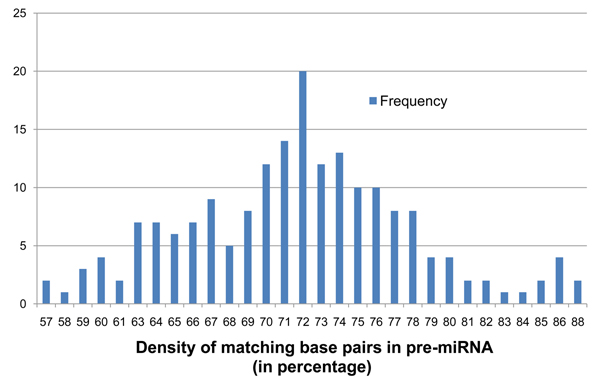
**Frequency distribution of parameter* D* on the 190 known pre-miRNA.** Shows the frequency distribution of densities of base-pair matches (*D*) between IRs detected on the 190 known pre-miRNA sequences of *Arabidopsis thaliana*. The values are rounded to the closest integer.

Since our initial target genome for functional IR identification will be a plant, criteria for distinguishing potentially functional from nonfunctional IRs were obtained from an analysis of 190 known miRNA precursors from *Arabidopsis thaliana*. The nucleotide sequences of these 190 pre-miRNA were retrieved from the miRBase database [17] and aligned against each of their reverse complements using irScan's variant of the SW algorithm. This generates the inverted repeat portion of the pre-miRNA that can be represented in the dot-bracket notation as shown in Figure 2 for the same secondary structure shown in Figure 1. It shows 52 matches among 63 nucleotide bases producing a relatively high 82.54% density of matches. This became our first criterion for miRNA detection. The density of matches in an IR, denoted *D*, generated from genomic DNA has to pass a predefined threshold, denoted *D_min_*, to be considered a sufficiently symmetric IR. The values of *D* seen among the 190 known pre-miRNA precursors ranged between 57% and 89%. To sufficiently reduce the number of IRs generated in the preliminary scan, we selected *D_min_* = 59% that excludes only 3 of the known 190 miRNA. It is important to note that the value of *D* for a pre-miRNA could be slightly different from what the IR detector finds for the same sequence in genomic DNA because the equivalent IR seen in genomic DNA could be a subset or a superset of the known pre-miRNA. Also, *D* can become 100% if the sequence contains a perfectly symmetric IR. This is never the case in pre-miRNA because loops in the hairpin are necessary for miRNA processing, but it is seen in low complexity regions of genomic DNA. We therefore also apply a *D_max_* of 95% to exclude such low complexity regions.  A frequency distribution of *D* on the 190 known pre-miRNA is shown in Figure 3.

Our second criterion of detection is based on the probability of occurrence of an IR in a randomly generated RNA sequence. Let us denote this as *P*. Small values of *P* most likely indicate highly conserved dyad symmetries and hence potential functionality, while large values of *P* most likely indicate a random RNA sequence. But they could also indicate a potentially functional RNA that has lost most of its symmetry but retained its functionality. Using *P* and *D* values as filters excludes such ncRNAs, but from our understanding of pre-miRNA processing, sufficient symmetry between inverted repeats is a necessary condition for the formation of stable hairpins that can be processed by the ribonuclease Drosha. The calculation of *P*, like *D*, depends on the ratio of matches to mismatches in the IR generated. This is described below.

Consider an RNA sequence with *2k* nucleotide bases. The left hand side (LHS) of length* k* bases is mostly inversely symmetric with the right hand side (RHS) of equal length resulting in the hairpin-like secondary structures shown in Figures 1 and 2. Let *n* be the number of bases that are inverted repeats (part of the base-pairing stem loop). The probability that *n* is exactly 1 is represented as *P(1, k) = 0.25 × (0.75)^k-1^ × k*, where *0.25* is the probability that one base in the LHS is the reverse complement of the corresponding base in the RHS, out of 4 possible bases A, C, G, or U. *(0.75)^k-1^* is the probability that all other *k-1* bases are mismatches. And *k* is the number of combinations in which this can occur. Similarly, if *n* is exactly 2, then *P(2, k) = (0.25)^2^ × (0.75)^k-2^ × ^k^C_2_*, where *^k^C_2_* is the number of combinations in which 2 matches can occur among *k-2* mismatches. In general, we can use the calculation below.

P(n, k) = (0.25)^n^ × (0.75)^k-n^ × ^k^C_n_

**Figure 4:**
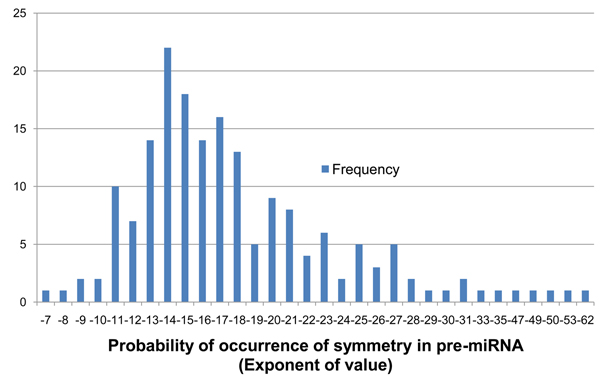
**Frequency distribution of parameter* P* on the 190 known pre-miRNA**. Shows the frequency distribution of the exponents of probability of occurrence of base-pair matches (*P*) between IRs detected on the 190 known pre-miRNA sequences of *Arabidopsis thaliana*. The values shown are the exponents of the probability value i.e. the exponent -11 indicates *P* values from 1.00×10^-11^ to 9.99×10^-11^.

The values of *P* among the known pre-miRNA ranged from 10^-7^ to 10^-62^. We selected 9.99×10^-9^ as *P_max_*, an upper bound threshold for *P*, which excludes two of the known pre-miRNA. A frequency distribution of *P* on the 190 known pre-miRNA is shown in Figure 4. Additionally, the values of P for various combinations of *n* and *k* were plotted and it was noted that *P* also reduced when *n* was much smaller than *k*, i.e. when there are many more mismatches than matching base-pairs in the IR. This was because the statistically most probable ratio of *n*:*k* is1:4 i.e. 25% of base-pairs in an IR are statistically most likely to be matches than mismatches in a randomly generated sequence. This follows from the fact that the nucleotide bases have an alphabet size of 4 (A, C, G, U). Hence, *P* tends to get smaller as this ratio becomes larger (or smaller) than 25%. With a combination of filters *D_min_* and *P_max_*, only the more symmetric IRs are detected. It can be argued that the two filtering criteria can be replaced with just *D_min_*, but the value of *P* is much more indicative of the statistical significance of an IR.

### Additional filters

**Figure 5:**
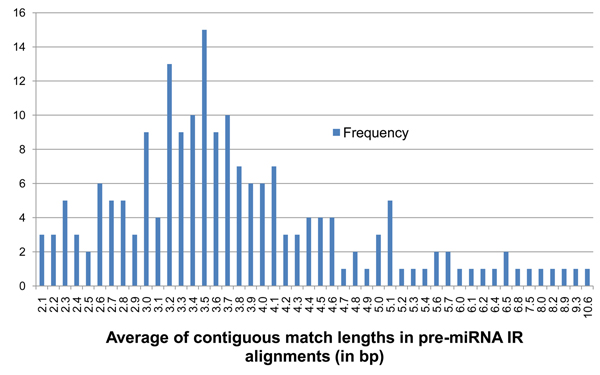
**Frequency distribution of parameter* A* on the 190 known pre-miRNA**. Shows the frequency distribution of average contiguous match lengths (*A*) for the 190 known pre-miRNA sequences of *Arabidopsis thaliana*. The values of *A* for each pre-miRNA IR has been floored to the closest number with 1 decimal place.

Using thresholds of D_min_ = 59%, D_max_ = 95%, P_max_ = 9.99×10^-9^, and a minimum IR length of 50 bp, irScan returns around 1.5 million IRs of which 186 match known pre-miRNAs. If duplicates are removed, this number goes down to 1.1 million with 183 known pre-miRNAs identified. To reduce this number further, additional filters are required. The third criterion for pre-miRNA detection was based on the observation that pre-miRNA secondary structures have relatively long stems. In the dot-bracket notation of Figure 2, these stems would be represented as contiguous matches. We calculated the average of contiguous match lengths in the IRs of the known pre-miRNA, denoted as *A*, and they ranged between 2.1 and 10.6 base pairs. For the IR in Figure 2, this is calculated as the average of lengths 6, 8, 1, and 11 making *A* = 6.5 bp. A base threshold of *A_min_* = 2.2 was selected which excluded 3 of the known 190 pre-miRNA. Any IR detected in genomic DNA with a value of *A* lower than 2.2 bp was filtered out. The frequency distribution of values of *A* seen on the 190 known pre-miRNA is shown in Figure 5.

**Figure 6:**
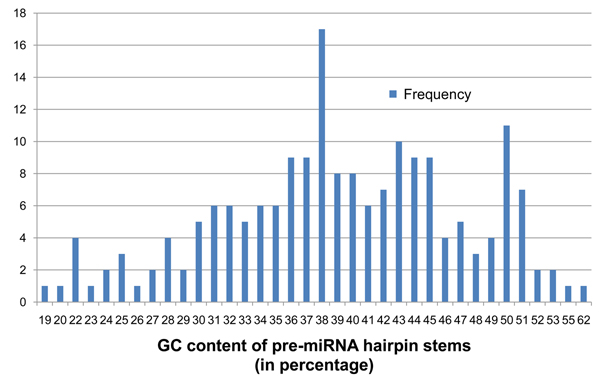
**Frequency distribution of parameter* G* on the 190 known pre-miRNA.** Shows the frequency distribution of GC content density (*G*) for the 190 known pre-miRNA sequences of *Arabidopsis thaliana*. The values of G for each pre-miRNA IR has been floored to the closest whole number lesser than it.

G-C base pairs in RNA sequences have three hydrogen bonds, making them more thermodynamically stable than A-U base pairs with two hydrogen bonds. Additionally, there is evidence that pre-miRNA hairpins are more thermodynamically stable than random sequences [18]. We therefore use GC content of the hairpin stems as the fourth criterion for pre-miRNA detection. The percentage of GC pairs in contiguous matches longer than 3 bp was calculated for each of the 190 known pre-miRNA. Contiguous matches that were 3 bp or shorter were more likely to belong to a loop than a stem in the hairpin-like secondary structure. So the GC content of these sufficiently long contiguous regions were calculated and denoted as *G*. For the IR in Figure 2, this is the percentage of GC pairs within the contiguous matching base-pairs of lengths 6 bps, 8 bps, and 11 bps. Among the 190 known pre-miRNA, *G* ranged from 18% to 62%. The base threshold *G_min_* was set to 18% that did not exclude any of the known pre-miRNA. The frequency distribution of values of *G* seen on the 190 known pre-miRNA is shown in Figure 6.

### The irScan framework

**Figure 7:**
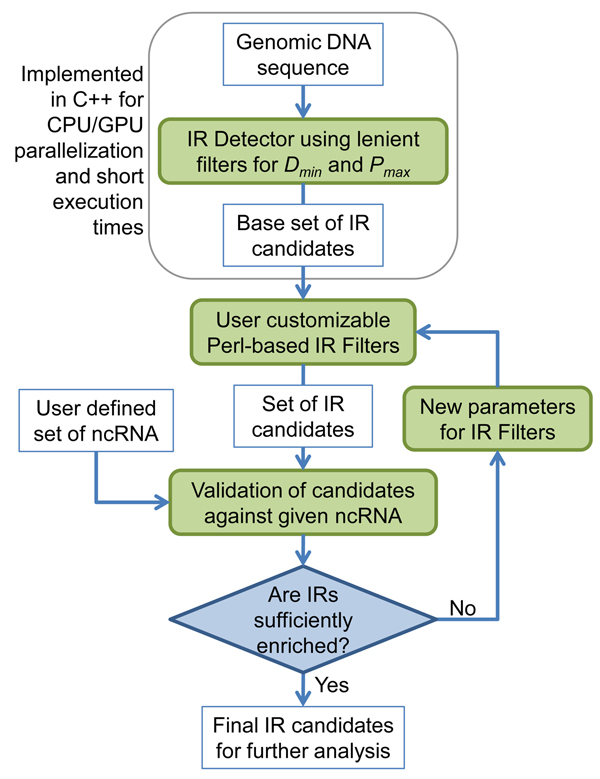
**The irScan framework for ncRNA identification**. The irScan software was designed and organized such that anyone with Perl programming knowledge could design their own filtering criteria for the base IRs detected by the irScan's C++ based IR detector that uses only the *D_min_* and *P_max_* (these are not specific to any particular type of ncRNA). Additional IR Filters are then user-defined in Perl and automated to find optimal parameter sets.

Figure 7 shows how irScan's software framework was organized so as to allow the identification of any type of ncRNA with the addition of new filters and requiring only Perl programming knowledge. The IR detector represents the most computationally demanding portion of the framework and was implemented in C++ to quickly produce a base set of IRs filtered using the preliminary base threshold values for *D_min_* and *P_max_*. The resulting large set of preliminary IRs detected could then be further enriched using customized filtering criteria coded in Perl. It was decided to use Perl to implement these additional filters because of its popularity among biologists and bioinformaticians. The parameters for these custom designed IR filters could be tested in a validation loop that continually tweak the parameters and rerun the validation until the IRs are sufficiently small in number for further analysis, while still retaining at least a predefined number of known ncRNA in the validation set.

## Results

### irScan using base parameters

The base parameters for irScan were selected to identify as many of the known 190 *At* pre-miRNA as possible, while keeping the total number of IRs detected less than 1 million. These parameters were *D_min_* = 59%, *P_max_* = 9.99×10^-9^,  A_min_ = 2.2 bp, and G_min_ = 18 bp. In all runs of irScan, *D_max_* was set to 95% to exclude low complexity regions, and IRs had to be at least 50 bp long to qualify as potential miRNA precursors. The irScan program returned 714700 IR candidates with these base parameters which included 186 of the known pre-miRNA sequences. If duplicate IRs generated by overlapping windows of different sizes were removed, then 483908 IR candidates remained with 183 of the known pre-miRNA sequences. Three of the initial 186 pre-miRNA were skipped because the simpler duplicate removal process cannot always distinguish between nearby identical IRs and duplicate IRs generated by overlapping scanning windows.

### Finding optimal parameters for irScan

**Figure 8:**
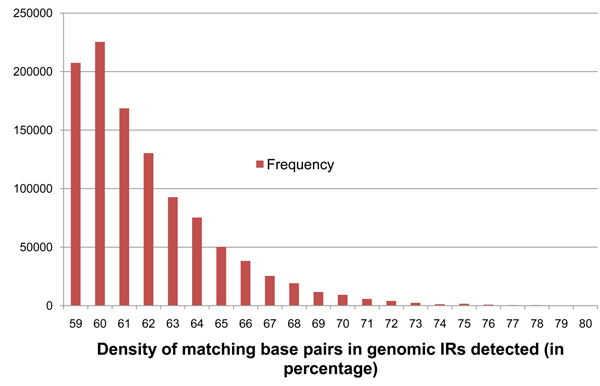
**Frequency distribution of parameter* D* on genomic *At* IRs**. Shows the frequency distribution of densities of base-pair matches (*D*) in IRs detected from genomic DNA of *Arabidopsis thaliana* with base thresholds of *D_min_* = 59% and *P_max_* = 9.99×10^-9^. The values are rounded to the closest integer.

**Figure 9:**
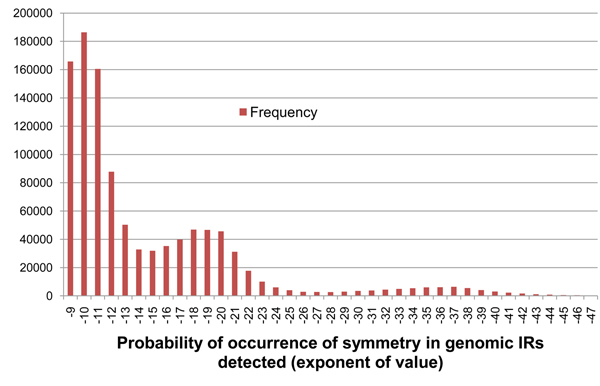
**Frequency distribution of parameter* P* on genomic *At* IRs.** Shows the frequency distribution of the exponents of probability of occurrence of base-pair matches (*P*) in IRs detected from genomic DNA of *Arabidopsis thaliana* with base thresholds of *D_min_* = 59% and *P_max_* = 9.99×10^-9^. The values shown are the exponents of the probability value i.e. the exponent -11 indicates *P* values from 1.00×10^-11^ to 9.99×10^-11^.

**Figure 10:**
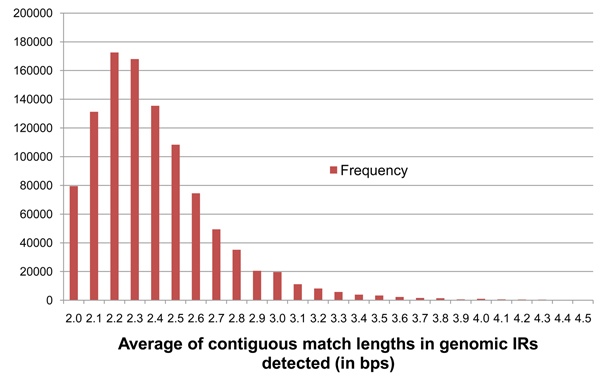
**Frequency distribution of parameter* A* on genomic *At* IRs.** Shows the frequency distribution of average contiguous match lengths (*A*) in IRs detected from genomic DNA of *Arabidopsis thaliana* with base thresholds of *D_min_* = 59% and *P_max_* = 9.99×10^-9^, *A_min_* = 2.0, and *G_min_* = 19. The values of *A* for each pre-miRNA IR has been floored to the closest number with 1 decimal place.

**Figure 11:**
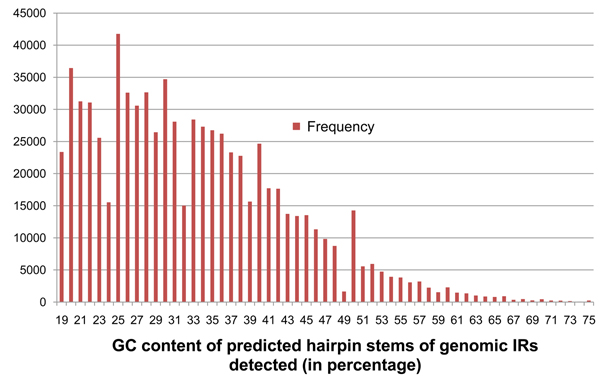
**Frequency distribution of parameter* G* on genomic *At* IRs.** Shows the frequency distribution of GC content density (*G*) in IRs detected from genomic DNA of *Arabidopsis thaliana* with base thresholds of *D_min_* = 59% and *P_max_* = 9.99×10^-9^, *A_min_* = 2.0, and *G_min_* = 19. The values of G for each pre-miRNA IR has been floored to the closest whole number lesser than it.

Optimal parameters for irScan was defined as those that generate the fewest IR candidates but still retained at least 90% of the 190 known pre-miRNA or 171 of them. IRs that were either a substring of a known pre-miRNA or that contained a known pre-miRNA sequence, were called Identified IRs (IIRs). A Perl script was written to repeatedly run irScan on a user-defined starting parameter set, find the number of IIRs identified, then increase or decrease the irScan parameters to identify closer to 171 IIRs, while minimizing the total number of IRs detected. This repetition was terminated if it found a set of parameters that identified exactly 171 IIRs, or if the user terminated the script when it was close enough to 171. Table 2 shows the IR and IIR counts for various combinations of parameters *A_min_* and *G_min_*. The values of *D_min_* and *P_max_* were fixed at 60% and  9.99×10^-11^ respectively, which by themselves retain around 94% of the known pre-miRNA (178 IIRs). Figures 8, 9, 10, 11 show the frequency distributions of all 4 parameters on the IRs detected on genomic DNA.

**Table 2:** Number of IRs and IIRs found using different irScan filters

	A_min_=2.2		A_min_=2.3		A_min_=2.4	
	**IRs**	**IIRs**	**IRs**	**IIRs**	**IRs**	**IIRs**

**G_min_=18**	260568	175	218041	175	169017	171
**G_min_=19**	251543	174	209739	174	161903	170
**G_min_=20**	243973	174	202877	174	156063	170
**G_min_=21**	232543	173	192640	173	147592	169
**G_min_=22**	222404	173	183721	173	140333	169
**G_min_=23**	211825	171	174453	171	132789	168
**G_min_=24**	201301	171	**165371**	**171**	125521	168
**G_min_=25**	193352	170	158493	170	119957	167
**G_min_=26**	180251	168	147397	168	111281	165

Shows the number of IR candidates generated by irScan on the 5 chromosomes of the Arabidopsis genome using various parameters. Parameters *D_min_* and *P_max_* were fixed at 60%  and 9.99×10^-11^ respectively while *A_min_* and *G_min_* were varied as shown. IIRs are the number of Identified IRs that uniquely match one of the known 190 pre-miRNA.

From Table 2, we can see that the optimal parameters were *A_min_* = 2.3 and *G_min_* = 24, with *D_min_* = 60% and *P_max_* = 9.99×10^-11^. This set of irScan parameters finds 165371 IR pre-miRNA candidates which include exactly 171 IIRs. This is still too large a number of candidates to warrant wet lab analyses on each, but it is a considerable reduction from the 1.5 million found by preliminary scans.

Recently, nine more Arabidopsis pre-miRNAs were added to miRBase. 6 of them matched one or more of the 165371 ncRNA candidates while the remaining 3 were just about insufficiently symmetric to pass the preliminary scans by the IR detector. They did not pass thresholds D_min_ and P_max_, but they did pass the A_min_ and G_min_ thresholds. This indicates that the selection of thresholds for symmetry were made slightly too stringent in an effort to keep the final set of candidates small. The user of the framework can tune these parameters to allow less symmetric sequences that compensate for more specific filters to find a smaller final set of candidates.

## Conclusions

Initially, our study revealed that partially symmetric inverted repeats are abundant in genomic DNA. However, it was shown that most of these IRs are easily distinguishable from the IRs of known pre-miRNA and can be filtered out using generic criteria like density of symmetry, statistical probability of symmetry, average length of symmetric regions, and the GC content of sufficiently symmetric regions. It is then reasonable to assume that more accurate filters that are highly specific to certain kinds of ncRNA will retain a smaller final list of IRs that can then be further analysed using wet lab techniques such as northern blotting to identify novel ncRNA genes. The irScan software framework was designed to be easily expandable with such additional filtering criteria, by anyone with experience in the Perl programming language. The more computationally demanding IR detector algorithm was implemented in C++ and parallelized to be able to scan the whole Arabidopsis genome for IRs in less than a minute using a base set of filters. A user could then filter these IRs further by running various combinations of filters using Perl to find an optimal set of filters and parameters, that minimizes the number of IR candidates while maximizing the number of known ncRNAs identified.

Additional filters are required to further enrich the final set of IRs with those that are more likely to be functional ncRNA, while still retaining most of the known ncRNA. Some such filters include the detection of promoters and terminators, homology analyses, location of candidate relative to coding regions or relative to each other, and better secondary structure prediction algorithms. Statistical analyses of related organisms can lead to filters for organisms that are less studied. The software developed is designed to easily accommodate such additional filters by someone with minimal experience in Perl, while the computationally expensive underlying genome-wide scanning algorithms have been implemented in the more efficient C++ programming language.

## Competing interests

The authors declare that they have no competing interests.

## Authors' contributions

CK participated in the conception and design of the study, developed all the software used for preliminary analyses, and designed and developed the Perl/C++ based irScan framework for ncRNA identification. RLF participated in the conception and design of the study and the biological aspects of miRNA analysis. FE participated in the conception, design, and the computational aspects of the irScan framework. All authors read and approved the final manuscript.
